# It might be a wonderful opportunity when patients with a psychotic disorder use cannabis

**DOI:** 10.1017/S0033291721003561

**Published:** 2022-03

**Authors:** Lieuwe de Haan

**Affiliations:** Amsterdam UMC, Amsterdam, Netherlands

There is nothing you can do to change several of the risk factors for an unfavorable course of psychosis. You cannot change the genes, the infections or stress during pregnancy, nor the neighborhood where people grew up, nor the trauma they suffered, but patients could stop using cannabis.

That would make a real difference because there is abundant evidence that people who have a psychotic disorder and stop using cannabis have a considerably better outlook, faster remission, less severe symptoms, less psychotic relapse, less readmission and improved social functioning (Linszen, Dingemans, & Lenior, 1994; Schoeler et al., 2016; Wade et al., 2006). Given the fact that about 26% of the patients with a psychotic disorder have a cannabis use disorder (Hunt, Large, Cleary, Lai, & Saunders, 2018) and about 33% of individuals with a first episode of psychosis use cannabis (Myles, Myles, & Large, 2016) and given the increasing availability of high potency tetrahydro-cannabinol products, interventions to reduce cannabis use are critical.

So, where are we waiting for? Or, is it too difficult for patients to stop? (Marconi, Di Forti, Lewis, Murray, & Vassos, 2016). Patients tend to continue their cannabis use. A Cochrane review of psychosocial interventions found little evidence that any type of therapy was more effective than treatment as usual (Hunt, Siegfried, Morley, Sitharthan, & Cleary, 2013). Why do interventions fail to make a difference? Are risk factors for cannabis use or dependence also difficult to change? Or do risk factors for cannabis use and psychosis overlap? Is the co-occurrence with nicotine addiction a complicating factor? Was the amount of money too little in the contingency management for cannabis use in an early psychosis trial (Sheridan Rains et al., 2019). Or are brief and optional modular interventions as in RAISE-EP simply not enough (Alcover, Oluwoye, Kriegel, McPherson, & McDonell, 2019)?

On one hand, we should not dismiss favorable findings. In most trials about 20–30% of patients stop using cannabis in both the intervention and control condition. More than 50% of the patients with a first psychotic episode who received psychoeducation stopped their cannabis use (Dekker et al., 2008). And even for persistent cannabis users there is some evidence from randomized controlled trials. Patients whose parents participated in a motivational interviewing training decreased their cannabis use at 3 month and 15-month follow-up, while patients whose parents received routine family support slightly increased their use (Smeerdijk et al., 2012, 2015). There are also indications that treatment with clozapine in those with persistent cannabis use could make a difference. In a randomized-controlled trial clozapine-treated patients reported a greater reduction in craving for cannabis, preserved processing of natural rewards and improved subjective wellbeing compared to risperidone treated patients. These improvements were associated with decreases in amygdala and insula activation during clozapine treatment (Machielsen, Veltman, Van den Brink, & De Haan, 2014, 2018). Moreover, in a cohort study, patients treated with risperidone reported significantly more craving for cannabis compared with patients treated with clozapine or olanzapine (Machielsen, Beduin, & Dekker, 2012). These differences between antipsychotics may be associated with differences in occupancy rate or dissociation rate of dopamine D_2_ receptors.

On the other, I propose that single interventions (motivational interviewing or family intervention or optimal medication) are not enough. The myriad of psychological and physiological factors underpinning dependence offers a substantial challenge. We probably need massive integrated intervention to reduce cannabis use in patients with a psychotic disorder. Persistent reduction in cannabis use may not be possible without changing daily activities, finding new friends, new hope, new goals, optimal medication and continuous support during a critical phase. We probably need to do it altogether. Because craving for cannabis is intense and associated with problematic daily functioning in most patients a specific modular intervention will not be sufficient.

Given the prevalence, the high THC content and the substantial influence on the course of psychotic disorders, the impact of cannabis reduction on the lives of people with psychosis can hardly be overestimated. Of course, we need high quality studies concerning intensive integrated interventions and concerning promising specific interventions like adding cannabidiol to regular treatment (Navarrete et al., 2021). And of course, the reciprocal influence of society and cannabis is complex. But in the meantime, I don't think we can afford the luxury of waiting with starting these integrated interventions until all the proof is provided.
